# Public needs for information disclosure on healthcare performance: Different determinants between Japan and the Netherlands: Erratum

**DOI:** 10.1097/MD.0000000000018402

**Published:** 2019-12-10

In the article, “Public needs for information disclosure on healthcare performance: Different determinants between Japan and the Netherlands”,^[1]^ which appears in Volume 98, Issue 43 of *Medicine*, the title in the citation information is missing the subtitle and should appear as:

Sasaki N, Groenewoud S, Kunisawa S, Westert G, Imanaka Y. Public needs for information disclosure on healthcare performance:Different determinants between Japan and the Netherlands. *Medicine*. 2019;98:43(e17690).
